# Factors associated with engagement and adherence to a low-energy diet to promote 10% weight loss in patients with clinically significant non-alcoholic fatty liver disease

**DOI:** 10.1136/bmjgast-2021-000678

**Published:** 2021-07-29

**Authors:** Jadine Scragg, Kate Hallsworth, Guy Taylor, Sophie Cassidy, Laura Haigh, Marie Boyle, Quentin Anstee, Stuart McPherson, Leah Avery

**Affiliations:** 1 Newcastle NIHR Biomedical Research Centre, Newcastle Upon Tyne Hospitals NHS Foundation Trust, Newcastle Upon Tyne, UK; 2 Nuffield Department of Primary Care Health Sciences, University of Oxford, Oxford, Oxfordshire, UK; 3 Translational and Clinical Research Institute, Newcastle University, Newcastle upon Tyne, UK; 4 Population Health Sciences Institute, Newcastle University, Newcastle upon Tyne, UK; 5 Centre for Rehabilitation, Teesside University School of Health and Life Sciences, Middlesbrough, UK

**Keywords:** non-alcoholic steatohepatitis, chronic liver disease, dietary factors, diet, obesity

## Abstract

**Objective:**

Clinical guidelines recommend weight loss to manage non-alcoholic fatty liver disease (NAFLD). However, the majority of patients find weight loss a significant challenge. We identified factors associated with engagement and adherence to a low-energy diet (LED) as a treatment option for NAFLD.

**Design:**

23 patients with NAFLD enrolled in a LED (~800 kcal/day) were individually interviewed. Transcripts were thematically analysed.

**Results:**

14/23 patients achieved ≥10% weight loss, 18/23 achieved ≥7% weight loss and 19/23 achieved ≥5% weight loss. Six themes were generated from the data. A desire to achieve rapid weight loss to improve liver health and prevent disease progression was the most salient facilitator to engagement. Early and significant weight loss, accountability to clinicians and regular appointments with personalised feedback were facilitators to engagement and adherence. The desire to receive positive reinforcement from a consultant was a frequently reported facilitator to adherence. Practical and emotional support from friends and family members was critically important outside of the clinical setting. Irregular working patterns preventing attendance at appointments was a barrier to adherence and completion of the intervention.

**Conclusions:**

Engagement and adherence to a LED in patients with NAFLD were encouraged by early and rapid weight loss, personalised feedback and positive reinforcement in the clinical setting combined with ongoing support from friends and family members. Findings support those identified in patients who completed a LED to achieve type 2 diabetes remission and highlight the importance of behaviour change support during the early stages of a LED to promote adherence.

**Summary box**

**What is already known about this subject?**

- Non-alcoholic fatty liver disease (NAFLD) is the most common liver condition worldwide.
- Clinical guidelines for the management of patients with NAFLD recommend lifestyle modification to achieve weight loss.
- A large proportion of patients with NAFLD find it challenging to achieve clinically meaningful weight loss.

**What are the new findings?**

- Patients with NAFLD are motivated to complete a low-energy diet (LED), despite the perceived difficulties with adherence to the intervention.
- The opportunity to achieve rapid weight loss, improve liver health and prevent NAFLD progression facilitated uptake and engagement with the LED. Adherence relied on early and rapid weight loss, personalised feedback from clinicians and positive reinforcement from a medical consultant. This emphasises the need for consultants to be aware of the progress made by patients undertaking the LED even though they typically would not be delivering this intervention to patients.

**Summary box**

**How might it impact on clinical practice in the foreseeable future?**

The LED delivered in secondary care has demonstrated to be an acceptable and feasible management option for patients with NAFLD. Should it demonstrate to consistently achieve long-term weight loss, it has the potential to reduce healthcare utilisation as people develop skills to effectively self-manage and could lead to deprescribing of medications. However, there are resource implications linked to the factors identified from the qualitative data reported:

- Engagement and adherence rely on a team approach to advocate the intervention and behavioural skills to actively support patients. As such, training is important to ensure that the clinical team provides a consistent message when advocating the LED and have sufficient knowledge and behavioural skills to provide ongoing support to patients.
- It is likely that a dedicated member of the clinical team would be required to provide behavioural support to patients undertaking the LED, particularly during the early stages of the intervention.
- Patient support groups could be beneficial to promote long-term engagement and adherence, particularly for those who are unable to attend frequent clinic visits or who do not have support at home.

## Introduction

Non-alcoholic fatty liver disease (NAFLD) is the most common liver condition worldwide, particularly in Western societies where it is estimated that 20%–33% of populations are affected.^1^ NAFLD is linked to increased energy consumption, limited physical activity and exercise and subsequent obesity. Overall, 40% of patients with NAFLD develop progressive liver fibrosis that may lead to cirrhosis and its associated complications in 5%–11% of individuals.^2 3^ Non-alcoholic Steatohepatitis (NASH)–cirrhosis is a common cause for liver cancer and an increasing indication for liver transplantation.^4^

In the absence of approved pharmaceutical agents, lifestyle modification, typically weight loss, is the cornerstone of NAFLD management.^5 6^ A weight loss goal of 10% of initial body weight is recommended for patients with clinically significant NAFLD^7^ as research has shown that 90% of patients who lose and maintain ≥10% weight loss resolve their steatohepatitis and 81% show improvements in fibrosis at 1 year.^7^ However, clinically significant weight loss and maintenance of weight loss remains a challenge. Research has shown that only 10% of patients maintain ≥10% weight loss after 1 year.^7^ Therefore, an acceptable intervention that can elicit significant, sustainable weight loss for patients with NAFLD would be advantageous.

Low-energy diets (LEDs) have proven to be a viable treatment strategy for people with type 2 diabetes mellitus (T2DM),^8^ enabling significant weight loss, with high levels of adherence and low levels of attrition in those patients with overweight or obesity.^9^ Furthermore, long-term weight loss maintenance in people with obesity has been associated with structured meal patterns with the absence of comfort eating.^10^ A randomised controlled trial of a LED delivered in primary care involving patients with T2DM (Diabetes Remission Clinical Trial (DiRECT)) reported that 24% of patients lost ≥15 kg, and mean body weight reduced by 10 kg at 1-year follow-up.^11^ It has been speculated that people with NAFLD might be less likely to engage with weight loss intervention due to NAFLD having a lesser symptomatic burden and perceived health risk when compared with T2DM.^12^ Despite the LED approach being used to actively reduce liver volume and fat prior to bariatric surgery since 2004, it had never been formally assessed as a treatment strategy for NAFLD until recently.^13–15^

Our study assessed the acceptability and feasibility of delivering a LED to patients with clinically significant NAFLD and found that it is a potential treatment option, with 34% of patients maintaining >10% weight loss at 9-month follow-up and 51% and 68% maintaining >7% and >5%, respectively.^13^

A recent systematic review synthesised qualitative studies investigating participants’ experiences of undertaking a Very low energy diet (VLED) achieved using total meal replacement products. The authors reported that VLEDs are well accepted by users and motivations to uptake included health-related outcomes and body image/appearance. Findings indicated that adherence was facilitated by rapid weight loss, group support meetings and ease of using the meal replacement products. Barriers to adherence were summarised as temptations and social occasions, and these were overcome by avoidance and distraction strategies.^16^ Similarly, a recent qualitative analysis of the Doctor Referral of Overweight People to a Low Energy total diet replacement Treatment (DROPLET) study reported that participants’ continuing relationship with the counsellor was an important part of adherence, in addition to the structured nature of the intervention.^17^ However, the acceptability of a LED to a population of patients with clinically significant NAFLD has yet to be qualitatively explored.

We conducted a qualitative interview study involving patients with clinically significant NAFLD who participated in a LED that aimed to initiate and maintain 10% weight loss.^13^ Specifically, our qualitative study aimed to identify factors associated with uptake, engagement and adherence to the LED intervention. In relation to adherence, we aimed to identify mediating factors that were both barriers and facilitators, including ways in which participants overcame barriers to adhere to the LED.

### Participants and intervention

A full description of the eligibility criteria and study schedule for patients participating in the LED feasibility study has been reported elsewhere.^13^ Briefly, patients with clinically significant NAFLD, aged ≥18 years with weight stability (±3%) were recruited from hepatology clinics at a tertiary centre in the UK (Newcastle upon Tyne Hospitals NHS Foundation Trust). All patients who completed the LED intervention (n=27) were invited to take part in an interview. The aim was to recruit a sample of patients to achieve maximal variation (eg, gender, those who struggled to adhere to the intervention and those who achieved maximal weight loss) and data saturation.

The intervention is reported in detail elsewhere.^13^ Briefly, it involved: a prescription of an 8–12-week LED (~800 kcal/day) consisting of meal replacement products (Optifast, Nestlè Health Science). Patients were encouraged to eat three portions of non-starchy vegetables and drink at least 2 L of water/calorie-free beverages each day. Demographic data and each individual participant’s home postcode were obtained during the baseline visit. The postcode was used to calculate the indices of multiple deprivation (IMD) Score and quintile.

### Qualitative data collection

Semistructured one-to-one interviews were conducted immediately following completion of the 8–12-week LED (n=23).^13^ All patients who completed the LED intervention were invited to a subsequent interview at 9-month follow-up (ie, following a 9-month period of weight loss maintenance); however, only four patients accepted the invitation (three declined the second interview as they did not speak English as their first language and 13 could not take part due to work commitments). All interviews were conducted by two members of the research team: a PhD researcher (JS) with expertise in lifestyle interventions in patients with liver disease and a chartered health psychologist (LA) with expertise in health behaviour change and qualitative research methods. Prior to the conduct of the interviews, one researcher (JS) delivered the intervention to all study participants, while the other (LA) had not met the patients. Patients could bring a friend or family member to the interview. Three patients elected to do this.

An interview topic guide (online supplemental material) was developed by the research team. Topics included motivations for taking part; expectations of the LED; perceived barriers to adherence and strategies used to overcome barriers and support requirements to maximise adherence. All questions were open ended and prompts were used to facilitate a more in-depth discussion to fully explore patient views. All interviews were audio recorded and transcribed verbatim.

10.1136/bmjgast-2021-000678.supp1 Supplementary data

### Methodological quality and reporting

The study was conducted in accordance with the consolidated criteria for reporting qualitative research to maximise methodological quality and transparency.^18^ To reduce social desirability bias, two researchers (one who had previously met with participants and another who had not) conducted interviews and analysed data (JS and LA) and three members of the research team (JS, KH and LA) interpreted data independently and agreed theme labels.

### Data analysis

Data were analysed using thematic analysis.^19^ To maximise trustworthiness (ie, rigour of the study relating to confidence in data and interpretation) of the findings, the following analyses procedure was undertaken: all interview transcripts were independently read and re-read by two researchers (JS and LA); both researchers independently coded segments of the data with reference to the first three interview transcripts to develop a coding strategy and generate preliminary themes/subthemes. Following discussion, the same researchers agreed a preliminary group of themes/subthemes. One researcher (JS) repeated this process with the remaining 20 interview transcripts and both researchers agreed a final set of themes and subthemes that best represented the data set following discussions. Disagreements were resolved by revisiting interview transcripts, discussing the text segments and when required asking the views of a third research team member (KH). Supporting direct quotes from patients were applied to the agreed thematic labels. The quantitative data analysis has been described in detail elsewhere.^13^ Briefly, primary and secondary data analyses were performed using IBM SPSS (V.24; IBM, New York, New York). Continuous data were tested for normality using the Shapiro-Wilk’s test, and data are presented as means±SD, unless otherwise stated. Correlations were measured using a Pearson correlation coefficient and differences between baseline and post VLCD were assessed using a paired sample t-test or a Wilcoxon signed-rank test where data were distributed non-parametrically. P value<0.05 was considered to be statistically significant.

All authors had access to the study data and reviewed and approved the final manuscript. This study was conducted prior to the COVID-19 pandemic and therefore all visits were conducted face to face, in person, however there is no reason why the behavioural support provided could not be conducted remotely by telephone or videoconferencing to support personal preference of patients and scalability. Although the conduct of outcome assessment may be more of a challenge (eg, blood testing).

## Results

Of the 30 patients who were enrolled into the study, 16 (53%) of all patients achieved ≥10% weight loss, 19 (63%) achieved ≥7% weight loss and 23 (77%) achieved ≥5% weight loss.^13^ Of the 30 patients who were recruited, 27 completed the LED and of those 23 were interviewed. Of the 23 patients who were interviewed, 14 (61%) achieved ≥10% weight loss, 18 (78%) achieved ≥7% weight loss and 19 (83%) achieved ≥5% weight loss. Patients attended an average of six appointments when completing the 8–12-week LED and percentage weight loss achieved was significantly associated with the number of appointments attended (r=0.569, p=0.001). Three patients dropped out or were withdrawn from the LED. One reported lack of family support to complete the diet, one reported an exacerbation of his depression meaning he could not attend study visits and one received a new diagnosis of cancer and was withdrawn. Interviews were offered to all three who withdrew from the study; however, these patients either declined or did not respond to the invitation. Overall, 23 patients (of 27 (85%) patients who completed the LED) agreed to take part in a semistructured interview. Interviews lasted between 15 and 46 min (mean: 28±9 min). The average age of patients was 56±11 years and average baseline body mass index (BMI) was 40±7 kg/m^2^ (table 1). The time between diagnosis of NAFLD and beginning of the LED ranged from 1 month to 9 years. The mean IMD Score was 29, with the majority of participants (n=8) falling within the 5th IMD quintile, indicating good representation of people from low socioeconomic status (SES) postcodes. These baseline characteristics closely matched those of the whole sample of patients taking part in the LED feasibility study, as confirmed by statistical analysis, showing good representation.^13^

**Table 1:** Baseline characteristics of qualitative study patients (n=23) and weight loss achieved immediately post low-energy diet

Variable	
Age (years)	56±11
Gender (male/female)	15/8
Body Mass Index (BMI) (kg/m^2^)	40±7
Weight (kg)	113±19
Time since NAFLD diagnosis (months)	
Mean	25±31
Median	12 (1–113)
Weight loss	
Absolute (kg) (mean)	14±13
Percentage weight loss (%) (mean)	12±5
Absolute (kg) (median)	12 (−2–24)
Percentage weight loss (%) (median)	12 (−1–20)
Achieved 10% weight loss (n)	14/23 (61%)
IMD	
Mean	29±20
Median	20 (5–75)
IMD quintiles (n (%))	
1 (least deprived)	3 (13)
2	5 (22)
3	4 (17)
4	3 (13)
5 (most deprived)	8 (35)

All data presented are mean±SD unless specified.

IMD, indices of multiple deprivation; NAFLD, non-alcoholic fatty liver disease.

**Figure 1:**
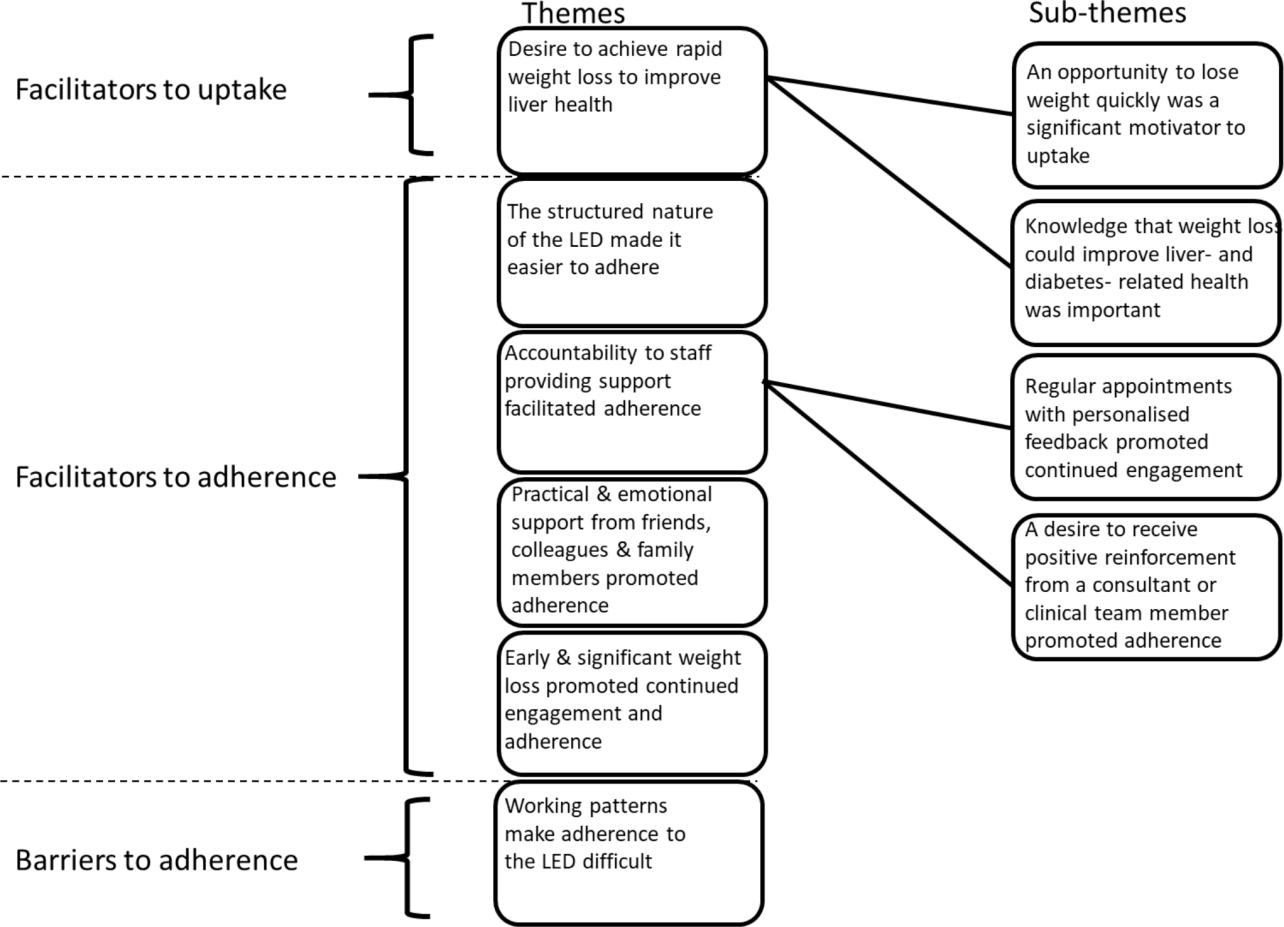
A summary of themes and subthemes generated from qualitative interview data. LED, low-energy diet.

The qualitative data collected from patients who took part in the LED intervention generated six themes and four subthemes that highlighted a number of factors associated with uptake, engagement and adherence. A summary of themes and subthemes is presented as figure 1, and table 2 presents a narrative summary of findings in relation to each theme and subtheme.

**Table 2:** A summary of themes and subthemes derived from thematic analysis of interview transcripts

Theme	Subtheme	Direct quotes
Desire to achieve rapid weight loss to improve liver health	An opportunity to lose weight quickly was a significant motivator to uptake Knowledge that weight loss could improve liver-related and diabetes-related health was important	‘The idea of quick weight loss…that appealed’ (male, aged 60 years. Weight loss achieved: 14 kg) ‘I think it was the fact that it was short term—quick and fast’ (female, aged 64 years. Weight loss achieved: 8 kg) ‘It wasn’t about vanity, you know? I just want to be healthier and live longer—that is what I am doing this for’ (female, aged 64 years. Weight loss achieved: 11 kg) ‘If I have eaten rich food you can feel a reaction almost from your liver. And I just wanted to feel better about that, I just wanted to feel healthier around that’ (male, aged 54 years. Weight loss achieved: 24 kg)
Accountability to staff providing support facilitated adherence	Regular appointments with personalised feedback promoted continued engagement The desire to receive positive reinforcement from a consultant or clinical team member promoted adherence	‘The fact that I am coming to see you on a weekly basis, or a fortnightly basis, it has kept me focussed’ (male, aged 41 years. Weight loss achieved: 15 kg) ‘Definitely, I think the main thing is the visits’ (male, aged 31 years. Weight loss achieved: 4 kg) ‘I suppose it’s a bit like being at school, isn’t it, and sort of saying, ‘when I go back and get weighed I want them to be pleased with me’’ (female, aged 55 years. Weight loss achieved: 11 kg) ‘I would have cheated without the support’ (male, aged 68 years. Weight loss achieved: 16 kg) ‘I like to come in every couple of weeks, just to, the talking is helping’ (male, aged 56 years. Weight loss achieved: 20 kg) ‘Seeing the surgeons face with a big smile. I walked in and he said ‘you’ve made my afternoon’’ (male, aged 56 years. Weight loss achieved: 20 kg)
The structured nature of the LED made it easier to adhere Practical and emotional support from friends, colleagues and family members promoted adherence		‘Not having to think about what to eat or what to cook. It made it so much easier because I’ve got such an erratic lifestyle’ (female, aged 55 years. Weight loss achieved: 11 kg) ‘It is more regimental. It is laid out clearly for me, and I can follow it easily. And with the advice I have been given as to what other bits of recipes I can do I found it very, very easy’ (male, aged 72 years. Weight loss achieved: 17 kg) ‘You do need a bit of your family to help you… if I was on my own, it would have been really, really hard’ (male, aged 61 years. Weight loss achieved: 12 kg) ‘The people I work with… they were really good, and they would bring food in, but they would eat it when I was away from the desk’ (female, aged 54 years. Weight loss achieved: 23 kg)
Early and significant weight loss promoted continued engagement and adherence		‘I didn’t think I would last in the first week… I got weighed, and then I’d lost all that weight in the first week, it gave me an incentive to continue with it’ (female, aged 54 years. Weight loss achieved: 23 kg) ‘In the first few weeks the motivation was seeing that I had lost a reasonable amount of weight pretty rapidly’ (male, aged 60 years. Weight loss achieved: 3 kg) ‘After the first initial five days, when I had lost all that weight, I was like, ‘Yes, this, this is working I can really do this’’ (female, aged 55 years. Weight loss achieved: 11 kg)
Working patterns make adherence to the LED difficult		‘If I was in a position where I could work nine till five or you know regular hours, same hours day after day, if I was in a good pattern I’d have no problem’ (male, aged 60 years’. Weight loss achieved: 14 kg) ‘I am really quite good during the week, unless I am having to work away and that makes it more difficult… I suffered where it was really difficult in work situations’ (male, aged 60 years. Weight loss achieved: 3 kg)

LED, low-energy diet.

### Desire to achieve rapid weight loss to improve liver health incentivised patients to take part

The opportunity to achieve rapid weight loss, specifically to improve liver health, prevent disease progression and improve control of their diabetes-related symptoms, was reported by patients as a significant motivator for the uptake of the LED. A minority (n=4) of patients reported a desire to lose weight to improve other health-related conditions including musculoskeletal pain and breathing difficulties that they felt were exacerbated by excess weight. It became apparent that advocacy of weight loss by a clinician, specifically a consultant, made use of a LED to achieve weight loss feel ‘more about health’ and ‘less about vanity’. Therefore, it was clear that health was an important motivator.

While the rate of weight loss was not specifically enquired about during interviews (see online supplemental material to view topic guide), the majority of patients (n=12) interviewed reported that rapid weight loss was more appealing than steady weight loss over a longer period of time. Many (n=13) reported having already tried a variety of weight loss approaches unsuccessfully; therefore, the offer of a rapid weight loss solution that could improve health and was supported by healthcare professionals facilitated uptake of the intervention.

### Accountability to team members providing support promoted adherence

Accountability to team members promoting use of the intervention and supporting adherence to it emerged as a common and salient facilitator to adherence (reported by 10 patients): *‘*When I go back and get weighed I want them to be pleased with me’ and ‘I would be saying to myself, whenever I have felt like picking, ‘no, I am going to see (member of the team)… you know?’ Specifically, accountability towards the initial referring member of staff was reported as a facilitator to adherence.

Regular appointments to discuss progress and receive personalised feedback were reported to be a key facilitator to adherence. Patients reported being motivated and encouraged by positive reinforcement from members of the clinical team.

### The structured nature of the LED made it easier to adhere

Ease of following the LED was frequently reported (n=15) as a facilitator to adherence. The meal replacements provided structure, removed decision-making around food choices and were reported as helpful to overcome the challenges of a busy lifestyle and as such was a facilitator to adherence. This theme was salient during interviews with all patients, irrespective of gender. Additionally, meal replacements provided flexibility for those who were required to travel for work and during holidays: ‘I just left a few of them at work so that I didn’t even have to remember to take them in with me’. Consistently the LED was reported as ‘simple’ and something that ‘didn’t require much thought’.

### Practical and emotional support from friends, colleagues and family members promoted adherence

A need for support outside of appointments was reported to be important to overcome everyday barriers to adherence. Nine patients reported work colleagues and family members making the diet easier to manage on a daily basis. Emotional support from family members was identified as having a major influence on adherence throughout the intervention. Other examples of practical, social and emotional support included family members restricting their own energy intake ‘My wife’s been the biggest supporter… she’s been eating the same amount of calories as me’ and ‘the help from my sister… we were always phoning each other up and she'd say, ‘Oh, I am starving.’ And I would say, ‘Oh just keep going, you will soon get over it.’…that helped’. Practical support was provided by family members who prepared suitable food for consumption post LED or monitored their family members adherence ‘They’ll say, give me a look at your book, to see if you’ve been cheating.’ And ‘she will go shopping and get me fresh prawns, fresh fish…more vegetables and buy stuff for stir-fry’s, get me water because I’m always drinking’’.

### Early and significant weight loss promoted continued engagement and adherence

Rapid weight loss, particularly during the first week of the intervention, was reported to be an important motivator for many patients (n=9) and a consistent facilitator to adherence. Patients reported that early weight loss kept them going throughout the challenging first week of the intervention. Patients also expressed surprise at seeing weight loss results so quickly and that the intervention had exceeded their expectations.

### Working patterns make adherence to the LED difficult

Barriers to adherence to the LED were highly individual. They included physically demanding jobs and family members providing food-related temptation. However, the most consistently reported barrier identified was irregular working patterns, reported by two patients. It became apparent that despite the structured nature and perceived flexibility of the intervention, patients who did not have a regular working pattern or those who could not leave work to attend study appointments struggled to adhere. Findings suggested that shift work and lack of support from the clinical team (due to non-attendance) hindered planning and patients did not develop skills to overcome challenges, for example, food temptations and the ability to deal with setbacks and fatigue.

### Weight loss maintenance

At the end of the 9-month post-LED follow-up period,^13^ four patients took part in a semistructured, one-to-one interview to explore motivators and facilitators to weight loss maintenance. Salient themes identified from these four interviews were very similar to those identified in association with the LED phase of the intervention. For example, patients reported accountability towards the clinical team and the need for social, practical and emotional support from clinicians (during appointments) and family members, friends and colleagues outside of clinical appointments to overcome everyday challenges. Patients reported shift work as a barrier to attending clinical appointments and as such did not feel that they had adequate support to maintain weight loss. Furthermore, they did not feel that they acquired the self-regulation skills required to maintain weight loss. Interestingly, patients reported inability to attend appointments due to work commitments as the primary reason for withdrawal throughout the post-LED follow-up period (weight loss maintenance phase), despite finding the intervention itself acceptable.

## Discussion

The LED for NAFLD was reported by patients to be acceptable and easier than expected to adhere to. Overall, 16 (53%) of all patients achieved ≥10% weight loss, 19 (63%) achieved ≥7% weight loss and 23 (77%) achieved ≥5% weight loss following completion of the LED.^13^ The qualitative data collected from patients generated six themes and four subthemes that highlighted a number of factors associated with uptake, engagement and adherence. The most salient facilitator to uptake was the desire to achieve rapid weight loss to improve liver-related and diabetes-related health and to prevent liver disease progression. Patients explicitly emphasised that they would not have been so willing to take part if the weight loss strategy offered was gradual over a longer period of time and that speed and magnitude of weight loss were of equal importance. While the level of weight loss achieved exceeded the expectations of participants, it also served as a facilitator to adherence beyond the initial stages of the VLCD intervention. Importantly, health rather than body image was the motivation for weight loss, highlighting the importance of communicating the specific health benefits of the LED in the context of NAFLD tailored to individual patient needs to promote uptake. While it is possible that patients reported health as the primary motivator for engaging with the intervention due to the overall aims and the setting of which it was based (hospital), they did report associations between weight loss and improved quality of life. Therefore, it is probable that patients were, in part, motivated to engage with the intervention due to health concerns. Future research should aim to explore in more detail why patients reported health as a motivator to uptake and to establish whether this was a consequence of the study being conducted in a hospital setting, or driven by information provided by HCPs.

Interestingly, 67% of patients offered this intervention were enrolled within 6 months from a single centre, compared with an uptake rate of 20% reported by the DiRECT trial.^20^ Although it is possible that our participants benefited from the widespread media coverage of the LED to achieve improvements in diabetes-related health, which may partly explain the greater uptake rate. Similarly, recruitment methods differed with our intervention being delivered in secondary rather than primary care. Encouragingly, findings suggest that patients with NAFLD are as motivated as patients with T2DM to engage with a LED intervention. It was previously thought that this might not be the case with a lot of patients with NAFLD being asymptomatic.

A significant strength of this study is the varied SES of participants who took part in the intervention. As reported in table 1, 35% of patients who were interviewed had a home postcode within the 5th (most deprived) quintile. Quantitative data from this study also demonstrated that we recruited patients with varying severity of NAFLD and multimorbidity.^13^ There was no defining demographic or clinical features of participants who withdrew from the study or who achieved a significant amount of weight loss, suggesting that this intervention has potential to be suitable to the needs of a wide range of patients with clinically significant NAFLD.

Factors associated with continued engagement with the intervention included accountability to the staff providing support. Patients highlighted that attending regular appointments with personalised feedback encouraged engagement. The number of appointments that patients attended was significantly associated with percentage weight loss achieved. Similarly, desire to receive positive reinforcement, specifically from their consultant, was very apparent and promoted adherence. Consultants promoting/endorsing the LED to patients also facilitated uptake. Despite the vast majority of participants reporting accountability to clinicians providing them with support, we did not specifically enquire about participants taking personal responsibility for adhering to the intervention, therefore this should be explored in future work. However, given the reported usage of self-management strategies to facilitate adherence to the intervention, it is likely that participants did feel a sense of accountability to themselves. With health being the primary facilitator to uptake, it is likely that this was also a facilitator to adherence, yet not explicitly reported. Future research should explore this in greater depth. The structured nature of the LED was reported to make adherence easier for the majority of patients. This finding supports other published research that has evaluated the use of a LED in people with obesity.^17 21^ This removed the decision-making process around food choices and was practically useful for work and some social events. Again, this finding supports previous studies.^17^ Outside of appointments, practical support from friends, family members and work colleagues was reported to be a salient facilitator to adherence, specifically to overcome temptation. Many patients reported that they might not have completed the LED without the support they received at home—one patient dropped out specifically due to the lack of family support to complete the LED. Early and significant weight loss was consistently reported to be linked to adherence, that is, those who achieved the greatest weight loss during the first 1–2 weeks were more likely to complete the intervention and this was reflected in the qualitative and quantitative data generated. This highlights the importance of providing intensive support during the early stages of the intervention to maximise weight loss and longer-term adherence to the intervention. This was also a finding of the DiRECT and DROPLET studies.^17 22^

As well as facilitators to uptake, engagement and adherence, a number of barriers were identified and generally resulted in patients dropping out of the intervention. These included irregular working patterns, in particular shift work. There is limited research evidence that has explored the impact of shift work on adherence to a LED. Therefore, it is not fully understood what it is about the LED that makes it unsuitable to shift workers, particularly when the meal replacement products used can be transported and consumed at any time. Shift work creates barriers to attending follow-up appointments where support is provided and this was reported in the current study. Therefore, our interpretation of this finding is that inability to attend appointments to receive support is a barrier to adhering to the LED. However, the suitability of the LED with shift workers should be explored further, particularly when considering the number of shift workers in the general population and the propensity of shift workers to be diagnosed with symptoms of the metabolic syndrome.^23^ While the LED was acknowledged to be flexible and easy to use in the workplace, the feedback and support provided during appointments were active intervention ingredients that impacted positively on adherence. Therefore, patients reported that the inability to attend appointments meant that they did not receive the emotional, practical and social support received by others. As such, it is possible that they did not acquire new self-regulation skills to overcome difficulties. Two other barriers were reported by a minority of patients and as such were considered to be unsubstantiated by the entire group. For example, two patients with physically demanding jobs reported feeling that the meal replacements did not provide sufficient energy requirements, that is, they experienced greater tiredness and fatigue than usual and this impacted negatively on adherence. Specifically, they reported that they felt tired at work and consequently struggled to carry out their usual activities. They reported that these feelings often led to consumption of food and/or drink while completing the LED in order to gain more energy. While family members and friends were considered to be a facilitator to adherence, they were also reported by one participant as a barrier by introducing temptation to foods. To overcome reported barriers, patients employed multiple behavioural regulation strategies including: behavioural goal setting, planning, food avoidance, including self-distraction and planning for difficult social situations. These strategies were positively reinforced during appointments which helped to embed them into the everyday lives of patients.

There are some overlaps with the findings from this study and others conducted previously in the context of lifestyle behaviour change. For example, it has been reported that physicians play a crucial role in the advocacy of lifestyle behaviour change interventions and their endorsement enhances uptake and adherence.^24^ This highlights the importance of the clinical team being knowledgeable and appropriately trained to effectively promote interventions and to provide positive ongoing reinforcement. It is also important that the messages communicated to patients about the potential benefits of interventions are consistent among clinical team members. Previous research has described the shared views of patients and clinicians with regard to the need for more information relating to their NAFLD diagnosis.^12 25^ Specifically, it was reported that the lack of engagement with lifestyle behaviour change may be a consequence of lack of awareness about NAFLD. In the context of this study, clinicians and patients expressed a preference for structured weight loss plans and patients expressed a desire to spend more time with a healthcare professional/clinician in order to gain a greater understanding of what NAFLD is and the role of lifestyle in disease management. Once this is achieved, it could be supported by a referral to specialist tier 3 weight management services where multidisciplinary teams use the LED regularly as part of their toolkit. Although effective pathways of referral would have to be established within NAFLD clinics.

Prior to agreeing to take part in the LED, the majority of patients had previously attempted to lose weight and maintain weight loss with varying levels of success. However, advice regarding weight loss for patients with NAFLD is often vague and unstructured and this was a finding of our previous work.^25 26^ As such, clinicians may benefit from training to improve the information they provide to patients to maximise the likelihood of behavioural change by targeting facilitators of behavioural intention including outcome expectancies and risk perceptions.^25 26^ Generally, awareness of NAFLD is low, even in populations at highest risk,^27 28^ therefore it is likely that knowledge about the role of weight loss on prevention of progression of NAFLD is lacking. Educating patients on the benefits of lifestyle change in the context of NAFLD management may improve uptake and adherence to a LED and other weight loss approaches. However, effectively educating patients on the benefits of lifestyle behaviour change to initiate and maintain weight loss relies on clinician advocacy. While education and information provision is not the primary method to drive motivation to change and other factors such as engagement with self-care, increasing this could serve to increase uptake and adherence to interventions.

When asked about ways in which the intervention could be improved to maximise engagement and adherence, several patients suggested that emotional and practical support from others undertaking the LED would be valuable. This finding has been reported previously in the context of a LED and T2DM.^29^

A further suggestion from patients was for clinicians to provide a summary of key clinical outcomes (eg, weight, BMI, blood pressure and HbA1c) to take home from each appointment to further increase motivation to adhere. This would help facilitate self-monitoring, subsequent self-management and allow patients to communicate progress to family members and friends or other healthcare professionals involved in their care. Personalised feedback throughout interventions is an evidence-informed strategy, particularly useful when paired with guidance on how to elicit further improvements.^30^ It came through in this study as vitally important to maximise engagement and adherence.

The dominant themes generated from this qualitative study overlap with those reported by previous qualitative evaluations of LED studies in clinical populations, including patients with T2DM and obesity.^29^ The salient motivators to uptake identified from our data support those reported in trials of LEDs undertaken in patients with T2DM; specifically, the desire to achieve rapid weight loss as an incentive to uptake and engagement with the LED.^29^

Overall, data support the LED as being acceptable to patients with NAFLD over an 8–12-week period, with limited data pertaining to weight loss maintenance at 9-month follow-up,^13^ and suggest that it has the potential to be a suitable treatment approach for some patients. In order to better understand the acceptability of the LED and barriers to engagement and adherence, it is important that this study is repeated with a more diverse cohort in terms of ethnicity and age. Furthermore, these data largely describe motivators, barriers and facilitators of participants over the initial 8–12 weeks of the intervention and does not adequately address long-term factors associated with weight loss maintenance. Although a proportion of participants approached to take part in a follow-up interview who declined reported using similar strategies during the maintenance period and as such did not feel that they had anything further to add. Future studies should aim to identify why patients refuse to take part in the intervention and explore specific barriers and problem-solving strategies for those who dropped out due to work commitments.

### Limitations

Interviews were primarily conducted following completion of the LED intervention. While a small number of interviews were conducted at the 9-month follow-up time period (n=4), we acknowledge that the data obtained were likely insufficient to adequately report on factors associated with maintaining long-term lifestyle behaviour change and this remains a challenge for the majority of people embarking on a LED intervention. Barriers to sustaining lifestyle behaviour change are multifactorial and include factors relating to individual financial circumstances, availability of social support and physical and psychological well-being.^31^ However, our primary aim was to assess the acceptability and feasibility of the LED for achieving 10% weight loss and identify factors associated with these aims, and in this regard we did achieve our aims. Future work should include an in depth exploration of the factors associated with long-term adherence in the NAFLD population. Given that the majority of patients declined a follow-up interview due to other commitments, including work, future studies could offer a remote means of interviewing and use strategies to emphasise the importance of better understanding barriers to long-term adherence. It could be argued that the frequency of communication between patients and members of the research team could have been a facilitator to adherence and it would be difficult to replicate this intensity of communication routinely in clinical care. Furthermore, the rapport developed between patients and members of the research team may have reduced the likelihood that patients reported negatively on the intervention. However, a second member of the research team not involved in intervention delivery assisted in the conduct of interviews to help overcome this issue. Similarly, lapses in adherence to the intervention may have been under-reported due to self-preservation bias.^32^ Patients who did not complete the intervention (n=3) were invited to be interviewed but did not consent. Therefore, these data reflect only the opinions of those who completed the intervention. Of those interviewed, not all achieved 10% weight loss (some achieved more, others less), therefore it could be of interest to specifically interview those who did not achieve this weight loss target to investigate whether the barriers and facilitators reported were similar to those who met the weight loss target. For the current study, analysing data relating to a subgroup of patients in this way would likely have required a larger number of patients to achieve data saturation and as such produce findings that are considered trustworthy. Furthermore, it would be beneficial in a future study to interview those who declined participation in the intervention. Finally, all patients within this study were Caucasian, and the views of patients from other ethnic groups were not explored. However, the sample interviewed was representative of those who took part in the intervention study, therefore future studies should aim to explore to increase uptake of a more representative NAFLD group.

While the withdrawal rate was low during the initial LED period (10%), withdrawal during the subsequent follow-up period was 33%, which is higher than that reported in other published studies including people with obesity and people with T2DM who are treated with insulin.^33 34^ The reasons for this are unclear and could relate to the differing patient populations or different study protocols, that is, delivery through referral to a specialised LED company compared with delivery through a secondary care clinical setting or the greater volume of study visits within this protocol. Future studies could explore ways to reduce withdrawal of participants during LED interventions by assessing the use of different mediums and modes of delivery.

A strength of the current study is that it benefited from the inclusion of a large proportion of typically ‘hard to reach’ populations, including those from lower socioeconomic backgrounds and middle-aged working-class men.^35^ Traditionally, this population can be difficult to engage in research and lifestyle interventions.^35^

### Conclusions

The use of a LED to achieve significant weight loss in patients with NAFLD is acceptable and feasible. Overall, patients found the intervention easier than anticipated to adhere to and rewarding—that is, it exceeded their expectations. While barriers were identified, further research is required in a larger, more diverse group of individuals with NAFLD to explore motivators, facilitators and barriers in more detail to develop effective strategies to elicit lifestyle behaviour change in clinical practice.

## Data Availability

Anonymised data are available upon reasonable request to the corresponding author.
