# Supplementary material for: Factors associated with engagement and adherence to a low-energy diet to promote 10% weight loss in patients with clinically significant non-alcoholic fatty liver disease
Source: BMJ Open Gastroenterol. 2021 Jul 29;8(1):e000678. doi: 10.1136/bmjgast-2021-000678 (PMC8323391; doi:10.1136/bmjgast-2021-000678)
Supplement: Supplementary data [file bmjgast-2021-000678supp001.pdf]

Is a very low calorie diet an acceptable therapy to achieve a target weight loss in patients with advanced non-alcoholic fatty liver disease?

Topic Guide for Patient Interviews (Semi-structured)

These interviews will investigate outcome expectations, general experiences, the challenges participants faced (including, for example, in their social and work environments), and approaches to overcoming them and adhering to the intervention at its different stages.

After 8-week Vlcd

- How did you find the diet? How did it compare to what you expected? What did you expect to get out of the diet?
- Was there anything you thought that might make the diet more difficult to stick to, before you started? (prompt: barriers with work, social events, holidays)
- What if anything made the diet difficult to stick to? (Prompt: any cravings, hunger, temptations, others eating foods you like, social events, holidays)
- How did you overcome these issues?
- What if anything made the diet easy to stick to?
- What advice would you give to others thinking about starting this diet?
- How have you found eating with friends/ family?
- What would make it easier?
- How did you fit this around work/ social events?
- How did you feel about the diet pre-intervention?
- Do you see the VLCD as the start of your weight loss? i.e, with a view to continuing via an alternative method?
- To what extent do you believe this diet had been effective?
